# Association of H-FABP with cardiovascular events: A systematic review

**DOI:** 10.34172/jcvtr.33039

**Published:** 2024-06-25

**Authors:** Ambili Parekumbel Venu, Rajamanickam Rajkumar, Divakaran Dinesh Roy, Sreelal Thekkumkara Prabhakaran, Kanagasabapathy Shankar, Vidhyadharan Jayapal, Sureka Varalakshmi, Sreeja Sreenivasan

**Affiliations:** ^1^Research Scholar, Meenakshi Academy of Higher Education and Research (MAHER-Deemed to be University), West K.K Nagar, Chennai, Tamil Nadu, India; ^2^Meenakshi Medical College Hospital & Research Institute, Meenakshi Academy of Higher Education and Research (MAHER-Deemed to be University), Kanchipuram, Tamil Nadu, India; ^3^Genetika Centre for Advanced Genetic Studies, Thiruvananthapuram, Kerala, India; ^4^Dr Moopen’s Medical College, Wayanad, Kerala, India; ^5^Hridayalaya Heart Foundation, Thiruvananthapuram, Kerala, India

**Keywords:** Heart-Type Fatty Acid-Binding Protein, Biomarker, Acute Coronary Syndrome, Coronary Artery Diseases, Acute Myocardial Infarction, H-FABP

## Abstract

The research aimed to evaluate the association between heart-type fatty acid binding protein (H-FABP) and cardiovascular events. We systematically reviewed research that has been conducted to assess this relationship, aiming to determine how useful H-FABP could be as a biomarker for cardiovascular diseases, especially in the initial phases of acute myocardial infarction (AMI) and acute coronary syndrome (ACS). Our goal was to validate its diagnostic accuracy and clinical relevance. We systematically searched through PubMed, Web of Science, and Google Scholar databases to find pertinent publications related to cardiovascular diseases and H-FABP, using various permutations, abbreviations, and language variations of MeSH keywords. The final analysis included 12 studies in total. The final study comprised twelve studies, and it was concluded that H-FABP demonstrated high sensitivity (64.3-91.5) and specificity (73-100) for diagnosing Acute Myocardial Infarction (AMI) and Acute Coronary Syndrome (ACS), especially within the first hours of symptom onset. H-FABP demonstrates potential in enhancing the overall diagnostic accuracy during the initial hours following the manifestation of symptoms. However, the existing data do not provide sufficient evidence to recommend the regular utilization of H-FABP as a preliminary risk assessment approach in individuals who present with suspected cardiac events. Additional investigations, with well-defined prospective cohorts, are needed to validate the results observed.

## Introduction

 For the past two decades, ischemic heart disease has remained the primary cause of death globally. It is responsible for 16% of the total deaths in the world. The death toll was 2 million in 2000; and it became 8.9 million in 2019.^1^ Identifying those at high risk is critical for implementing timely prevention strategies and thereby lowering the burden of CVD.

 Cardiac biomarkers are crucial in the diagnosis of cardiovascular diseases. Acute myocardial infarction is typically diagnosed utilizing the diagnostic schema outlined by the World Health Organization (WHO). This includes the existence of a clinical history characterized by chest pain, variations in the ECG and serum enzyme findings.^2^ The early detection of AMI can be challenging due to ambiguous changes in electrocardiogram (ECG) and delay in the release of cardiac enzymes, including as Creatine Kinase Isoenzyme MB (CK-MB).

 The identification of a swiftly developing biochemical marker that is unique to myocardial damage would enable the implementation of a more suitable diagnostic and therapeutic approach in individuals who are suspected of having AMI and experiencing chest pain. A perfect marker capable of predicting the onset of the disease could help to reduce deaths due to CVD. Several biomarkers have been studied for their ability to predict cardiovascular events, including heart-type fatty acid-binding protein (H-FABP). The potential of this biomarker to indicate myocardial damage was first recognized in 1988.^3^ H-FABP is a small cytosolic protein that is very prevalent inside the myocardium. It is often referred to as mammary-derived growth inhibitor. The FABP3 gene, situated on the 1p33-p32 fragment of chromosome 1, is responsible for encoding H-FABP.^4^ Long-chain fatty acids are transported with the help of H-FABP. It is not purely cardio specific, since it’s also present in skeletal muscle, brain, and kidneys.^4^ The release of H-FABP into the bloodstream occurs as a result of myocardial injury, and it can be detected from a blood sample.^4^ H-FABP serum levels in healthy people are typically in the single digit ng/ml range.^5-7^ The microRNA miR-1 controls the expression of H-FABP and may contribute to the development of heart failure itself.^8^ Rapid release of H-FABP from myocytes into the systemic circulation is seen subsequent to myocardial damage. This release is attributed to the diminutive dimensions and unbound cytoplasmic distribution of H-FABP. Moreover, it is postulated that temporary elevations in sarcolemmal membrane permeability may facilitate the release of H-FABP into the bloodstream.^9,10^ Even following short-term ventricular stress, this “wounding” of myocytes was found, and it is likely to have substantial impact in different autocrine and paracrine pathways involved in pathogenesis of the heart failure.^9^

 Following the initial observation that H-FABP is released from isolated rat hearts exposed to ischemia followed by reperfusion,^11^ the first indications of the potential diagnostic value of H-FABP for ischemic heart disease was emerged in early 1990s. Two separate research groups demonstrated that the concentration of this biomarker exhibited a significant and early increase after acute myocardial infarction.^12,13^ The H-FABP assay has been developed as both a quantitative and qualitative method and has undergone evaluation in a significant number of diagnostic cohort studies and several studies have indicated a potential link between H-FABP levels and cardiovascular events, making it a promising biomarker for risk evaluation of cardiovascular events. The objective of this systematic review is to examine the research conducted to investigate the correlation between H-FABP and cardiovascular events.

## Materials and methods

###  Literature search

 In June 2023, a comprehensive search was performed on electronic databases such as PubMed, Google Scholar, and Web of Science to identify relevant papers. This search included several variations, acronyms, and language variations of the keywords: ‘cardiovascular disease’, ‘coronary heart disease’, ‘atherosclerosis’, ‘coronary syndrome’, ‘myocardial infarction’, ‘coronary artery disease’ and ‘heart-type fatty acid-binding protein’. In addition, we conducted a manual search of the reference lists of pertinent reviews in the field to find additional articles for inclusion. The literature search had no language restrictions. The scope of our investigation was limited to peer-reviewed articles that focused on human adult participants.

###  Review Method and Selection Criteria

 Studies were considered suitable for inclusion in the analysis if they demonstrated a correlation between H-FABP and the incidence of cardiovascular events. We selected and included studies that provided data on H-FABP in patients with ACS/AMI/CAD/CVD as well as cohort studies where H-FABP was tested after ACS/AMI/CAD/CVD and tracked longitudinally. Additionally, cross-sectional studies and case-control studies were included.

###  Data Extraction

 The data from each study were extracted and summarized, and summary tables were created. Data extracted from all studies includes: Publication year, lead author, study population, numbers of cases with cardiac events and control subjects without events, mean age, sex, subjects with DM, subjects with hypertension, current smoking, study design, H-FABP assay methods, sensitivity, specificity, and data from ROC curve.

## Results

**Figure 1:**
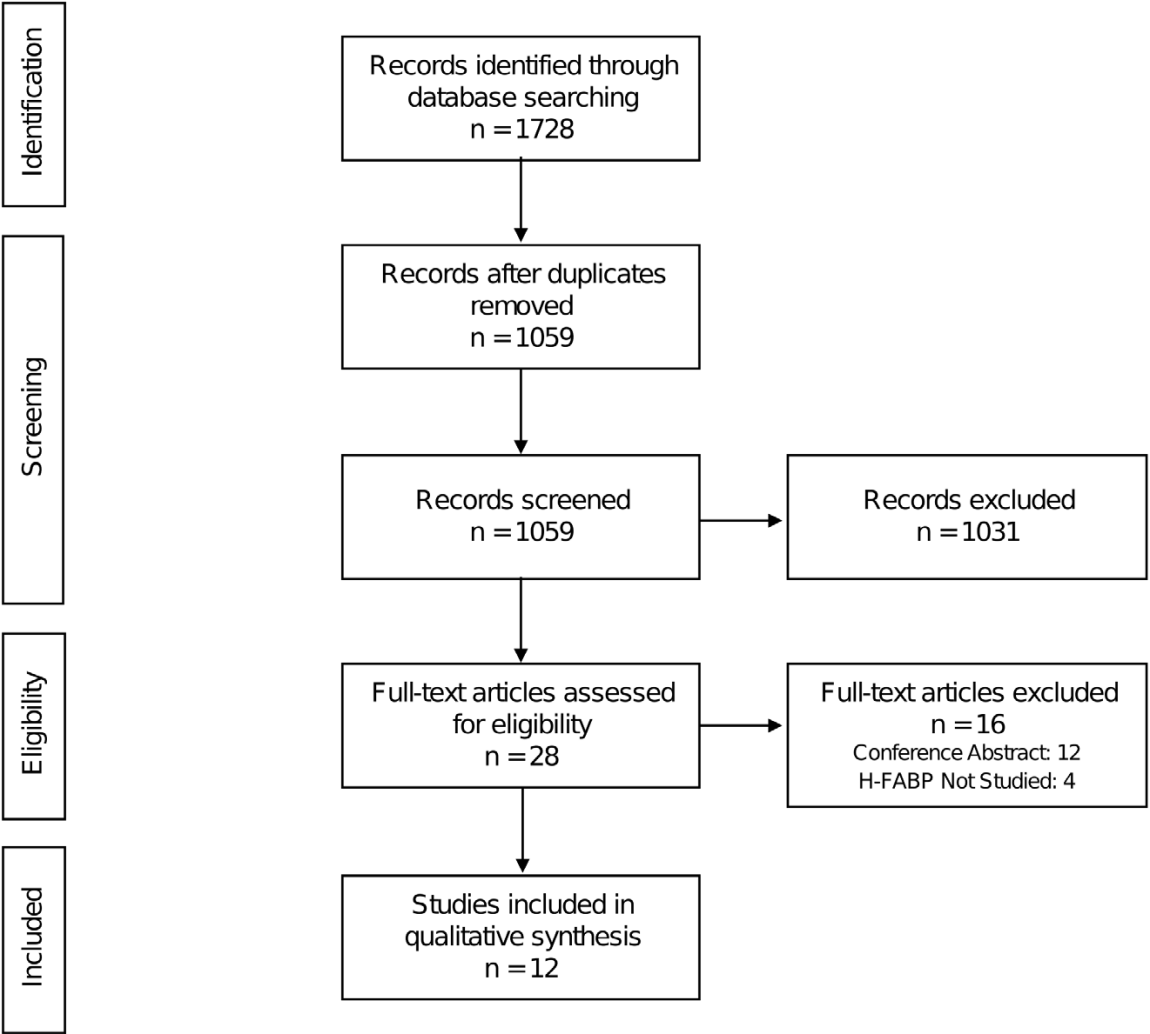
PRISMA flow chart of the study selection process

 The systematic search of three electronic databases and examination of research citations yielded 1728 entries in total. After removing the duplicates, 1059 records remained. 1031 records were excluded after thorough review of abstracts. 16 studies were further eliminated based on the established criteria. The systematic review included the remaining 12 original papers (Figure 1). Table 1 shows the baseline characteristics of the studies that were included in the analysis.

**Table 1:** Baseline characteristics of the included studies

**Sl. No.**	**Author**	**Year**	**Study Type**	**Population(n)**	**Sample Size**		**Age in Years Mean (SD)**		**DM (%)**		**HTN (%)**		**Smoking (%)**	
					**Events**	**Non-Events**	**Events**	**Non-Events**	**Events**	**Non-Events**	**Events**	**Non-Events**	**Events**	**Non-Events**
1	C. Geraldine McMahon^14^	2010	Cohort Study	Patients brought to ED with chest pain of possible cardiac origin (1128)	117	1011	64	52	10.3	7.4	43.6	33.5	47.9	42.0
2	Mi-Gil Moon^15^	2021	Cohort Study	Patients presented to the ER within 24 hours following the onset of chest pain and/or dyspnea (89)	65	24	61	65.8	23.1	20.8	49.2	45.8	44.6	20.8
3	Atindra Narayan^16^	2022	Cohort Study	Patients having chest pain with suspicion of AMI (50)	36	14	46.9	46.9	NR	NR	NR	NR	NR	NR
4	Le Xuan Truong^17^	2020	Descriptive cross-sectional study	CVD patients (179), Healthy Controls (57)	179	57	61	NR	NR	NR	NR	NR	NR	NR
5	Robert TA Willemsen^18^	2019	Cross-sectional study	Participants with chest pain or discomfort who had consulted a GP (303)	32	271	58.3		7.3		NR		NR	
6	Saba Raza^19^	2022	Cohort Study	Patients presented to an ED or admitted to the CCU with symptoms suggesting angina (154)	111	43	60	55	64	46.5	75.7	55.8	28.8	14
7	Atay^20^	2019	Cohort Study	Patients admitted to the ED with sudden onset chest pain and who were diagnosed with ACS (60)	60		63.6		NR		NR		NR	
8	Karakus Yilmaz Banu^21^	2014	Prospective and cross-sectional study	Patients admitted to ED with chest pain within first 48 hours and suspected ACS (66)	36	30	58.8		88	93.3	58.3	43.3	NR	NR
9	Yanling Miao^22^	2020	Prospective and cross-sectional study	Patients with myocardial infarction (44), healthy subjects (45)	44	45	54.6	54.6	NR	NR	NR	NR	NR	NR
10	Hui-Wen Zhang^23^	2020	Prospective cohort study	Patients underwent coronary angiography (CAG) due to angina-like chest pain (4370)	353	4017	59.9	58.1	57.2	54.7	72.8	64.9	24.9	31.3
11	Markus P. Schneider^24^	2022	Prospective cohort study	Patients with moderately severe CKD (5217)	5217		60.5		36.2		NR		15.4	
12	Hui‐Wen Zhang^25^	2020	Prospective cohort study	Patients underwent coronary angiography (CAG) because of angina-like chest pain (4594)	380	4214	60.1	58.0	55.3	39.8	72.6	64.8	25	31.9

CAD: coronary artery disease, CAG: coronary angiography, DM: diabetes mellitus, HTN: hypertension, MI: myocardial infarction, NR: not reported; SD: standard deviation.

 The final analysis included a total of 16356 patients. Among these, 6630 patients (40.5%) were eventually diagnosed with a cardiac event. Patients with AMI were included in five studies.^14-17,22^ Patients with ACS were included in four studies.^18-21^ Twostudies enrolled patients with CAD.^24,25^ One study enrolled patients with CVD.^23^ The trials were set in the Emergency Department in six studies.^14,15,17,19-21^ Two studies included patients undergone CAG due to angina like chest pain.^23,25^ Eight studies were cohort studies,^14-16,19,20,23-25^ while the rest were cross sectional studies.^17,18,21,22^ Two studies exclusively studied H-FABP,^18,25^ while rest of the studies used multiple biomarkers such as cTnI,^14-17,19-23^ CK-MB,^14-17,20,21^ MYO,^14,21^ TnT,^24^ and Gal-3.^20^ The studies used various methods to analyze H-FABP levels. One study used Randox Laboratories’ cardiac array (biochip).^14^ Every individual biochip enables the concurrent and quantitative identification of MYO, cTnI, CK-MB and H-FABP from a 50 μL specimen. Two studies used latex turbidimetric immunoassay (LTIA) method.^15,25^ Chromatographic immunoassay was carried out in one of the studies.^16^ Turbidity immunoassay on fully automatic Mindray BS800M was performed in another study.^17^ Quantitative Fluorescence immunoassay method was also used in a study.^19^ Commercial enzyme-linked immunosorbent assay (ELISA) kits are used in one study.^20^ Rapid chromatographic immune test designed for qualitative determination of H-FABP was used in one of the studies.^21^ Beckman Coulter AU5800 automatic biochemical analyzer was used to perform Enzyme-linked immunosorbent sandwich assay in another study.^22^ Hitachi 7180 chemistry analyzer was used to perform turbidimetric immunoassay for one study.^23^

###  H-FABP against Acute Myocardial Infarction

 Following are the five studies which evaluated H-FABP for the detection of AMI: Geraldine McMahon,^14^ Mi-Gil Moon,^15^ Atindra Narayan,^16^ Le Xuan Truong,^17^ and Yanling Miao.^22^ The confirmation of AMI in the studies were made using predefined criteria.^26,27^

 Geraldine McMahon tested the efficacy of H-FABP, cTnI, myoglobin and CK-MB for the detection of AMI in patients brought to the ED with chest pain of cardiac origin.^14^ Multiple blood samples were collected during admission and then intervals of 2 hours up to12 hours and then at 24 and 48 hours. Biomarkers were measured using Randox Laboratories’ cardiac array. The diagnostic cut-offs (99%, CV < 10%) were - H-FABP: 5.24 μg/L, MYO: 95.57 μg/L, cTnI: 0.37 μg/L, and CK-MB: 7.18 μg/L. Highest sensitivity was demonstrated byH-FABP at early time points. 64.3% till 3 hours and 85.3% from 3 to 6 hours among the biomarkers investigated. Between 0 and 12 hours after the onset of chest pain, H-FABP showed the highest NPV (93%-98%). Furthermore, this research additionally investigated the diagnostic efficacy of each marker on the cardiac array at each time point. According to the ROC curves, it was shown that H-FABP had the greatest diagnostic accuracy among the four markers within the time period of 0 to 12 hours after the onset of pain.

 Mi-Gil Moon conducted laboratory investigations and point-of-care testing (POCT) for H-FABP, CK-MB and cTnI for patients who were brought to the ED within 24 hours after experiencing chest pain and/or dyspnea and referred for suspected ACS.^15^ A venous blood sample was first obtained in the ED for the purpose of conducting laboratory analysis and POCT. Subsequent samples were obtained for conducting sequential laboratory examinations on cardiac markers. Latex turbidimetric immunoassay (LTIA) method was used for the Laboratory analysis of H-FABP. Rapid kits using immune chromatography (ICA) method were employed for the POCT of cardiac markers. Among all the patients, the observed AUC values are as follows: H-FABP - 0.771(95% CI, 0.674–0.868), TnI - 0.759(95% CI, 0.664–0.854), and CK-MB -0.690(95% CI, 0.580–0.801). H-FABP exhibited the highest AUC value, and there was no statistically significant difference between H-FABP and TnI (*P* = 0.81, Delong’s test). Additionally, the patients were categorized based on the time of their ER visit after the onset of symptoms. Among the patients presented at the ER within 4 hours, only the AUC value of H-FABP was above 0.7, which is 0.738 (95% CI, 0.591–0.885). There was no statistical difference with TnI (*P* = 0.37, Delong’s test). The AUC values for H-FABP, TnI, and CK-MB in the group of patients who arrived at the emergency room within 4 to 24 hours were 0.835 (95% CI, 0.712–0.957), 0.883 (95% CI, 0.760–1.0), and 0.805 (95% CI, 0.671–0.939). There was no statistically significant difference between H-FABP and TnI (*P* = 0.81, Delong’s test). The diagnostic accuracy for AMI was as follows: H-FABP - 56% (95% CI, 45–67), TnI– 37% (95% CI, 27–48) and CK-MB - 35% (95% CI, 25–46).

 Atindra Narayan conducted a study on 50 individuals who were suspected of having AMI and were admitted to the hospital within 12 hours of experiencing chest pain. Blood samples were collected while hospitalizing and also after 24 hours.^16^ A chromatographic immunoassay was performed to examine the level of H-FABP using a direct sandwich ELISA kit. The study found that the H-FABP level in the blood sample of AMI patients was more than the level of non-AMI patients and normal subjects. Cut off levels were as follows: H-FABP - 7.9 ng/ml, Troponin - 8.2 ng/ ml and CK-MB – 25U/I. In normal subjects, the average level of H-FABP was 2.8 ng/ml and in the confirmed AMI patients, it was 123 ng/ml. For the patients having non-AMI with chest pain, 7.9 ng/ml was the H-FABP level. The cut off level of H-FABP shows the highest determination efficiency of H-FABP. Comparison of sensitivity of H-FABP, CK-MB and troponin showed that H-FABP has high sensitivity. Specificity comparison showed that specificity of H-FABP is less than others. The study showed that the diagnostic effectiveness of H-FABP and Troponin is similar.

 Le Xuan Truong conducted a cross-sectional study among 179 AMI patients who were treated in the ED and 57 healthy control subjects. H-FABP was tested using turbidity immunoassay.^17^ The study showed that the concentration of H-FABP increases age increases. The finding correlates to few others previous studies as well.^28-30^ The concentration of H-FABP increased very early at 0-3 hours with a median concentration of 30ng/ml. Then the concentration quickly peaked after 6-12 hours at 169.8 ng/ml and gradually decreased after 12-24hours. However, CK-MB had a median concentration of 28.6 U/L at 0-3 hours, then at 6-12 hours it gradually increased to 45.3 U/L. Then slightly decreased to 44.9 U/L at 12-24 hours.When compared to H-FABP and CK-MB, TnI concentrations appeared more slowly in the blood after 3 hours with a median concentration of 0.1 ng/ml at 3-6 hours, then it increases from 6 hours and peaks at 12-24 hours. Higher sensitivity was exhibited by H-FABP than CK-MB and TnI in the time intervals 0 to3 hours, 3 to 6 hours, and 6 to 12 hours. After 12 to 24 hours, the sensitivity of troponin I was higher than that of CK- MB and H-FABP. Between 0 to 24 hours, combining all 3 tests together demonstrated a highest sensitivity of 97.2%. In terms of specificity, H-FABP always reached 100% and was always higher than troponin I and CK-MB between 0-24 hours when all the 3 tests are combined.

 Yanling Miao studied an observation group of 44 patients with MI and control group of 45 healthy subjects. H-FABP levels and cTnI levels of the subjects were analysed and compared.^22^ Enzyme-linked immunosorbent sandwich method was used to determine H-FABP levels. It was observed that the H-FABP and cTnI levels in the observation group were markedly higher when compared the control group (*P* < 0.05). The study compared biomarker levels across different grades of cardiac function, revealing a sequential increase in H-FABP and cTnI levels among patients with MI as the grades of cardiac function increased. H-FABP and CTnI levels were as follows: CFG I: 4.64 ± 1.16, 0.92 ± 0.18, II: 4.94 ± 1.22, 3.15 ± 0.61, 9.38 ± 1.82, 8.63 ± 1.74, IV: 9.83 ± 1.96, 12.78 ± 2.54.

###  H-FABP against Acute Coronary Syndrome

 Four studies evaluated H-FABP for the detection of ACS: Robert TA Willemsen,^18^ Saba Raza,^19^ Atay E,^20^ and Karakus Yilmaz Banu.^21^

 Robert TA Willemsen has studied 303 patients who has consulted a General Practitioner (GP) and presented with chest pain.^18^ A point-of-care (POC)H-FABP test with was performed for the participants (cut-off value 4 ng/ml). For ACS, the study reported a sensitivity of 25.8% for the POC H-FABP test. 91.6% was the reported NPV for ACS. Also, the test failure reported was 4.0%. Positivity was 5.3%. The study found that the diagnostic value of a point-of-care H-FABP test when used alone was inadequate, leading to a significant occurrence of false negative test outcomes.

 Saba Raza conducted cross-sectional study of 154 patients brought to the ED with symptoms suggesting angina.^19^ A quantitative fluorescence immunoassay analyzer was used to measure H-FABP levels. The observed median level of H-FABP was 17.8 ng/ml and it was 0.113 ng/ml for hs-TnI. In subjects with ACS, the median levels of both biomarkers were markedly higher than in subjects without ACS (*P*value < 0.001). H-FABP demonstrated a diagnostic accuracy of 87.58%. Among the 154 patients, there were 101 true positive cases, 10 false positive cases, 10 false negative cases, and 33 true negative cases when a cutoff value of ≥ 7 ng/ml for H-FABP was specified. Better diagnostic performance was exhibited by H-FABP in patients with chest pain for ≤ 3 hours. The AUC of H-FABP was 0.911(95%CI, 0.850-0.972) and hs-TnI was 0.908 (95% CI, 0.844-0.971) was observed in Subjects who had chest pain < 3 hours exhibited a high sensitivity of 98.3%, however the specificity was 22.7%, the cutoff of level being 5 ng/ml. An overall sensitivity of 91.0% and specificity of 76.7% was shown by H-FABP. 90.9% and 76.7% were the PPV and NPV values respectively.

 Atay E studied 60 patients who were brought to the ED and presented with chest pain.^20^ The participants were classified into three groups: individuals with unstable angina pectoris, individuals with MI accompanied by acute ST segment elevation in ECG, and those with MI without ST segment elevation in ECG. Venous blood sample was collected at admission, followed by further samples at 2nd and 4th hour after admission. Commercial ELISA kits were used to measure H-FABP and GAL-3 levels and solid-phase radial partition enzyme immunoassay was used for cTnI. The study showed that, at the time of admission, as well as at the second and fourth hours, there were statistically significant differences between the groups in terms of the levels of H-FABP. These differences emanated from the NSTEMI group (*P* = 0.001, *P* = 0.003, and *P* = 0.003, respectively). Patients in NSTEMI group had lower levels of H-FABP when compared to patients in the STEMI group. This is most likely because the NSTEMI group was admitted to the ER earlier, which resulted in less myocardial damage. The study revealed a significant positive relation between cTnI and H-FABP levels upon admission (*P* = 0.022), as well as a moderate positive relation between cTnI and H-FABP levels in the second hour in patients diagnosed with USAP (*P* = 0.040). There was no significant change seen in the levels of CK-MB, cTnI, and H-FABP at the 4th hour in patients with USAP (*P* > 0.05). Additionally, no significant difference was seen in the H-FABP levels between patients with USAP at admission and the 2nd and 4th hours (*P* > 0.05). There were no statistically significant differences seen in the H-FABP levels of patients with STEMI at admission, the 2nd hour, and the 4th hour (*P* > 0.05).

 Karakus Yilmaz Banu studied 66 patients came to the ED, presented with chest pain and suspected ACS.^21^ Blood samples were collected from the subjects and investigated forH-FABP, Myoglobin, Troponin and CK-MB levels. Rapid chromatographic immune test method was used to measure H-FABP. In 15.2% patients, H-FAPB values were positive and it was negative in 84.8% patients. The study found a statistically significant change in all biochemical markers between individuals with and without ACS (*P *values of 0.015, 0.004, 0.012, 0.008 for CK-MB, Myoglobin, cTnI, and H-FABP, respectively). The addition of H-FABP to commonly utilized biomarkers resulted in an improvement in sensitivity, specificity, PPV, and NPV values. However, this increase did not reach statistical significance. AUC values for H-FABP and cTnI were 0.61 (95% CI, 0.48–073) and 0.51 (95% CI, 0.52–0.76), respectively. Furthermore, it is shown that H-FABP attains its peak concentration during a span of 4 hours following the occurrence of cardiac damage. Positive H-FABP readings were seen in 10% of patients who were admitted to the ED within 4 hours after experiencing chest pain, and in 31.3% of patients who were admitted to the ED longer than 4 hours. There existed a statistically significant difference in the positivity of H-FABP between admissions within the first 4 hours and admissions beyond 4 hours (*P* < 0.05).

###  H-FABP against CAD and CVD

 Three studies studied H-FABP for the detection of CAD/CVD: Hui-Wen Zhang,^23^ Markus P. Schneider,^24^ and Hui‐Wen Zhang.^25^

 Hui-Wen Zhangenrolled 4370 angiography-proven CAD patients in the study.^23^ Latex immunoturbidimetric method was used to measure H-FABP. The reference interval was < 5 ng/mL. In total subjects, the mean H-FABP level was found to be 2.45 ng/mL. During a median follow-up period of 51 months, it was noted that 8.1% of patients experienced CVEs. The number of cardiovascular fatalities observed was 69, whereas 43 cases of nonfatal MI, 90 cases of stroke, and 151 cases of coronary revascularization, encompassing both PCI and CABG, were recorded. The study found that individuals with CVEs had substantially higher levels of H-FABP (2.83 ± 2.27 ng/mL) compared to those without (2.38 ± 1.72 ng/mL). Additionally, the study utilized Kaplan-Meier curves to illustrate that the individuals in the upper quartile of H-FABP had the highest likelihood of experiencing overall cardiovascular events and cardiovascular fatalities. Furthermore, the Cox proportional hazards model reveals a positive correlation between elevated levels of H-FABP and an increased risk of CVEs. After controlling for several factors such as age, gender, family history of CAD, hypertension, dyslipidemia, diabetes, current smoking, and HbA1c, the participants with higher levels of H-FABP continued to exhibit a significant and independent correlation with poorer cardiovascular outcomes. Additionally, it was noted that individuals with elevated levels of H-FABP had a notably heightened susceptibility to cardiovascular mortality (*P* = 0.033 in the Pre-DM group, *P* = 0.002 in the DM group).

 Markus P. Schneider examined a cohort of 4951 individuals who were participants in the German Chronic Kidney Disease (GCKD) study. The investigation involved the measurement of H-FABP and hs-TNT levels. The determination of 24 H-FABP was conducted with an enzyme-linked immunosorbent assay. This study employed Cox proportional hazards models to investigate the correlations between H-FABP and hs-TNT with non-CV mortality, CV mortality, MACE, and hospitalization for CHF throughout the follow-up period. Throughout the study’s follow-up period, there were 579 deaths related to non-CV causes, 190 deaths related to CV causes, and 522 and 381 hospitalizations for CHF, respectively. The research revealed that across all four examined outcomes, the Hazard Ratios associated with H-FABP were comparatively lower than those associated with hs-TNT. The study also observed that there was no statistically significant correlation between H-FABP and the occurrence of CHF hospitalizations after adjusting that data for hs-TNT (Hazard ratio, 1.64 [95% CI, 0.90-2.99]).

 Hui‐Wen Zhang conducted a study on 4594 individuals who had coronary artery disease confirmed by angiography.^25^ Latex immunoturbidimetric method was used to measure H-FABP levels. The study posited that those exhibiting elevated H-FABP levels were of advanced age in comparison to those displaying lower H-FABP levels. There was no statistically significant variation in H-FABP levels based on gender distribution (*P* = 0.499). Additionally, it was observed that individuals exhibiting elevated H-FABP levels demonstrated a greater prevalence of hypertension and diabetes, as well as elevated levels of glucose, creatinine, HbA1c, and decreased eGFR, in comparison to patients with lower values. The study further indicated that those with heightened H-FABP levels exhibited a higher Gensini score and a greater occurrence of CVEs. In addition, the levels of H-FABP were substantially higher in individuals who experienced an event compared to those who did not experience an event (mean: 2.95 ng/mL versus 2.41 ng/mL, *P* < 0.001).

## Discussion

 This systematic review emphasizes the heterogeneity of the results regarding the associations between H-FABP and cardiovascular events described in the literature at hand and there are several aspects which remains inconclusive. Furthermore, even though a thorough search strategy was employed, only 12 relevant studies were found that fit the criteria for inclusion and exclusion.

**Figure 2:**
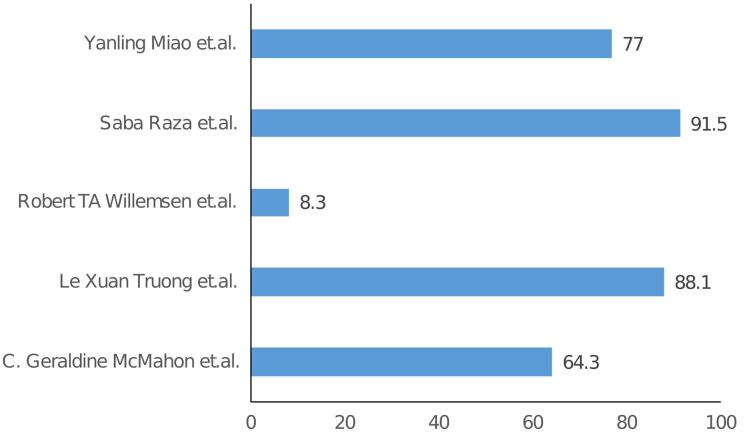
Sensitivity of H-FABP

**Figure 3:**
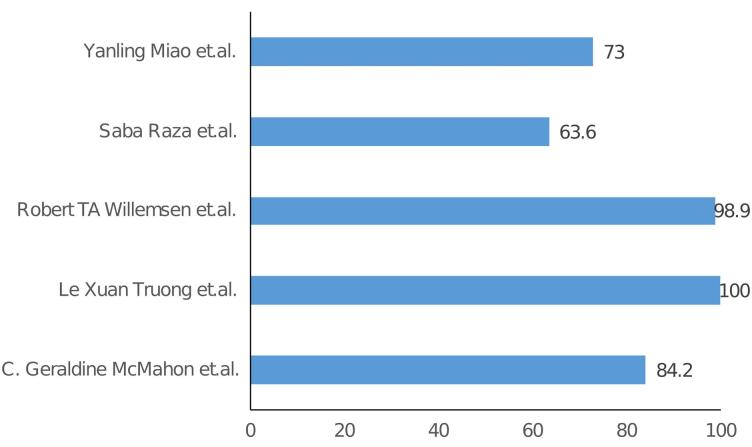
Specificity of H-FABP

 The specificity and sensitivity of H-FABP from the included studies are listed in Table 2 and Table 3, and graphical representation is shown in Figure 2 and Figure 3 respectively. In the studies examining the correlation between H-FABP and diagnosis of ACS, various biomarkers such as CK-MB, Tn and MYO demonstrated comparatively high specificities. It has been also observed that the specificity of H-FABP is lower when compared with Tn and CK-MB.^16^ However, their diminished sensitivities within the initial 6-hour period following the manifestation of symptoms limits their utility in the early diagnosis of ACS. However, highest sensitivity was observed for H-FABP during the early time points. This could be attributed to several factors, such as the elevated levels of HFABP in the myocardium in comparison to other tissues, its relative tissue specificity,^31^ stability and solubility, low molecular weight and the rapid release into plasma subsequent to myocardial damage–approximately sixty minutes following an ischemic attack.^32^ Also, one study indicated that the H-FABP levels in plasma were below the threshold for normal patients but over the threshold for verified AMI and non-AMI subjects.^16^ The H-FABP cut-off level exhibits its highest determination efficiency. It has been also revealed that H-FABP and troponin showed similar diagnostic efficacy.^16^ Additionally, it should be noted that the peak of H-FABP occurred prior to that of CK-MB or troponin T. Furthermore it has been observed that H-FABP demonstrates superior performance as a diagnostic marker in differentiating acute STEMI from non-AMI conditions compared to troponin. H-FABP levels were shown to rise sharply between 0 and 3 hours, peak between 6 and 12 hours, and then decline gradually between 12 and 24 hours. This shows that H-FABP is a very important part of diagnosing AMI, especially in the early stages. Another study revealed that H-FABP has a sensitivity of 91.5% for individuals experiencing chest pain lasting three hours or fewer. This sensitivity surpasses the sensitivity of troponin I, which is 83.0%. This finding emphasizes the significance of high sensitivity in promptly identifying the presence of the illness. In contrast, the specificity of H-FABP was lower than that of hs-TnI. The specificity of H-FABP in a group of patients who had chest pain three hours after its beginning was found to be 90.5%, which was similar to the specificity of hs-TnI (91%). In terms of test performance, H-FABP and hs-TnI were evaluated using AUC and ROC. The results showed that H-FABP had a higher AUC in patients with chest pain lasting less than three hours, followed by hs-TnI. The utilization of H-FABP has been found to enhance the sensitivity, specificity, PPV, and NPV values of commonly employed biomarkers, hence increasing the probability of achieving a correct diagnosis. However, it is important to note that this enhancement did not demonstrate statistical significance.^21^

**Table 2:** Sensitivity of H-FABP, CK-MB, MYO and cTnI

**Author**	**% Sensitivity**															
	**0-3 h**				**3-6 h**				**6-12 h**				**12-24 h**			
	**H-FABP**	**CK-MB**	**MYO**	**cTnI**	**H-FABP**	**CK-MB**	**MYO**	**cTnI**	**H-FABP**	**CK-MB**	**MYO**	**cTnI**	**H-FABP**	**CK-MB**	**MYO**	**cTnI**
C. Geraldine McMahon et al^14^	64.3	39.3	39.3	50	85.3	58.8	61.8	68	89.9	75.9	65.8	81	90.1	87.3	50.7	95.8
Le Xuan Truong et al^17^	88.1	59.5		14	91.2	62.6		9.9	96.4	71.4		78.6	83.3	83.3		94.4
Robert TA Willemsen et al^18^	8.3															
Saba Raza et al^19^	91.5															
Yanling Miao et al^22^	77			75												

**Table 3:** Specificity of H-FABP, CK-MB, MYO and cTnI

**Author**	**% Specificity**															
	**0-3 h**				**3-6 h**				**6-12 h**				**12-24 h**			
	**H-FABP**	**CK-MB**	**MYO**	**cTnI**	**H-FABP**	**CK-MB**	**MYO**	**cTnI**	**H-FABP**	**CK-MB**	**MYO**	**cTnI**	**H-FABP**	**CK-MB**	**MYO**	**cTnI**
C. Geraldine McMahon et al^14^	84.2	95.8	95.8	93.3	88.7	96.2	93.9	94.3	93.5	98.1	96.3	94.2	91.4	97.9	95.8	94.3
Le Xuan Truong et al^17^	100	93		94.7	100	93		94.7	100	93		94.7	100	93		94.7
Robert TA Willemsen et al^18^	98.9															
Saba Raza et al^19^	63.6															
Yanling Miao et al^22^	73			76												

 Study examining the levels of biomarkers across several grades of cardiac function revealed a decline in LVEF among patients with myocardial infarction (MI) in grades III and IV, as compared to grades I and II.^22^ The values of LVEDD, LVESD, LVESV, and E/A exhibited an increase. There was a progressive increase in the levels of H-FABP and cTnI in individuals with myocardial infarction (MI) as the cardiac function grades rose. These findings suggest that the utilization of LVEF, LVEDD, LVESD, LVESV, E/A, cTnI, and H-FABP levels is beneficial in assessing the categorization of cardiac function in patients with MI.

 When patients are classified as USAP, STEMI and NSTEMI, there are statistically significant differences in the levels of H-FABP at admission, 2nd and 4th hours (*P* = 0.001, *P* = 0.003, and *P* = 0.003, respectively).^20^ It is evident that the NSTEMI group is the source of the differences. H-FABP levels were low in the NSTEMI group of patients. The observed phenomenon can be attributed to the early admission of patients to the ER, as well as the relatively lower extent of myocardial injury observed in patients with NSTEMI in comparison to those with STEMI.

 The diagnostic utility of a point-of-care (POC) H-FABP test as a standalone test was inadequate. The sensitivity of the test for acute coronary syndrome (ACS) was found to be 25.8%, resulting in a significant occurrence of false negative test outcomes.^18^ The study proposed an algorithm capable of effectively predicting the end of ACS based on the presence of a sensation of pressure on the chest, the lack of left lateral chest chain, shortness of breath, ST elevations, ST depressions and the results of the H-FABP POC test result. Nevertheless, as compared to the clinical judgment of a GP, the benefits were limited. While the false negative rate experienced a reduction of 50.0%, there was a corresponding rise of 47.7% in the over-referral of patients that are ultimately ACS-negative.

**Figure 4:**
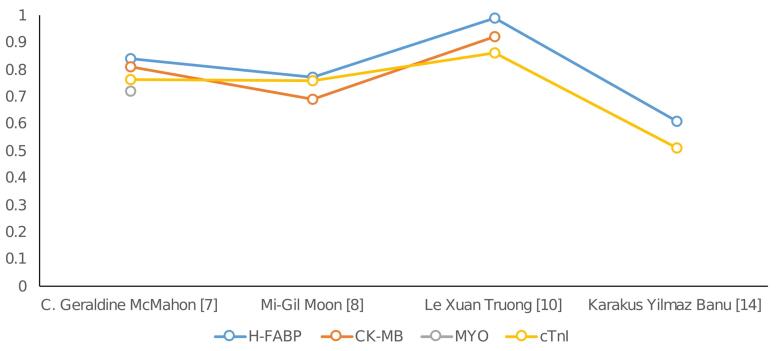
Comparison of Area under the ROC curve from different studies

Figure 4 presents a comparative analysis of the Area under the ROC curve across several studies. H-FABP consistently ranks first in nearly all studies that include AOC data. A comprehensive examination of ROC curves indicates that H-FABP had the highest level of diagnostic accuracy compared to other markers within the timeframe of 0 to 12 hours after the onset of pain.^14^ Among the biomarkers studied, H-FABP has the highest NPV for detecting AMI. The observation of this discovery occurred within a 12-hour timeframe following the onset of chest pain, suggesting that HFABP is a reliable indicator to rule out MI.

 Some studies assessed the prognostic value of H-FABP.^15^ H-FABP demonstrated the ability in predicting the degree of left ventricular dysfunction after MI. But, it did not excel in determining the extend of CAD. Not with standing its comparatively low correlation coefficient, it is noteworthy that only the early level of H-FABP demonstrated a statistically significant association with LVEF and WMSI (*P* = 0.038). This observation suggests that H-FABP exhibits the property of being detectable during the first phase of myocardial damage. A further finding pertained to the progressive exacerbation of coronary artery stenosis in individuals exhibiting increased levels of H-FABP.^23^ Additionally, it was found that individuals who experienced cardiac events exhibited significantly elevated levels of H-FABP in comparison to those who did not experience cardiac events. Furthermore, patients in the top H-FABP group exhibited a rate of adverse cardiac events that was approximately 1.5 times greater than those in the lowest H-FABP group. Subsequent analysis employing the Cox proportional-hazards model revealed a significant association between patients with elevated levels of H-FABP and unfavorable outcomes. Another study’s data indicated that people with poor glucose metabolism exhibited increased levels of H-FABP.^25^ It is worth noting that there was an independent association between H-FABP and worse clinical outcomes in patients with CAD who had pre-DM or DM. It is also suggested that the increased levels of H-FABP in the bloodstream could potentially act as a prognostic marker for unfavorable outcomes in patients with CAD who have mild abnormalities in glucose metabolism.

 Following are the strengths of this systematic review. It summarizes the existing data on H-FABP levels in different cardiovascular event conditions and evaluates the diagnostic accuracy of H-FABP compared to other biomarkers. The prognostic value of H-FABP in predicting future cardiovascular events is assessed in the study. It also indentifies the potential limitations of current research on H-FABP as a biomarker for CVEs.

 The limitations of the study are as follows. The levels of H-FABP may be influenced by renal function due to the predominant renal excretion of low-molecular-weight proteins such as H-FABP,^33,34^ consequently, persons with renal failure would see a reduction in H-FABP levels. Nevertheless, this constraint also poses a restriction for cardiac troponin (cTn). Muscle injury can also lead to false positive results due to the presence of H-FABP in both cardiac and skeletal muscles. However, it is important to note that the H-FABP concentration is significantly elevated in the heart compared to muscles.^32^ Additionally, it has been documented that a significant proportion of patients, approximately 30%, who exhibit elevated levels of cardiac troponin (cTn) do not exhibit the typical symptoms associated with ACS.^35^ This systematic study included one study which only included patients with renal failure while majority of rest of the studies excluded such patients.^24^

## Conclusion

 The utilization of heart-type fatty acid-binding protein (H-FABP), both as an independent marker and in combination with other biomarkers, demonstrates potential in enhancing the overall diagnostic accuracy during the initial hours following the manifestation of symptoms. Nevertheless, the existing evidence is presently impacted by the substantial heterogeneity observed among the various studies. Hence, it is important to exercise caution when interpreting the findings of this systematic review. However, the existing data do not provide sufficient evidence to recommend the regular utilization of H-FABP as a preliminary risk assessment approach in individuals who present with suspected cardiac events. Additional investigations, with well-defined prospective cohorts, are needed to validate the results observed.

## Competing Interests

 Authors declare no conflict of interests in this study.

## Ethical Approval

 The present study is based on published data, and hence ethical approval was not required.
